# Digital Healthy Diet Literacy and Self-Perceived Eating Behavior Change during COVID-19 Pandemic among Undergraduate Nursing and Medical Students: A Rapid Online Survey

**DOI:** 10.3390/ijerph17197185

**Published:** 2020-09-30

**Authors:** Tuyen Van Duong, Khue M. Pham, Binh N. Do, Giang B. Kim, Hoa T. B. Dam, Vinh-Tuyen T. Le, Thao T. P. Nguyen, Hiep T. Nguyen, Trung T. Nguyen, Thuy T. Le, Hien T. T. Do, Shwu-Huey Yang

**Affiliations:** 1 School of Nutrition and Health Sciences, Taipei Medical University, Taipei 110–31, Taiwan; sherry@tmu.edu.tw; 2 Faculty of Public Health, Hai Phong University of Medicine and Pharmacy, Hai Phong 042–12, Vietnam; pmkhue@hpmu.edu.vn; 3 President Office, Hai Phong University of Medicine and Pharmacy, Hai Phong 042–12, Vietnam; 4 Department of Infectious Diseases, Vietnam Military Medical University, Hanoi 121–08, Vietnam; nhubinh.do@vmmu.edu.vn; 5 Division of Military Science, Military Hospital 103, Hanoi 121–08, Vietnam; 6 Institute of Preventive Medicine and Public Health, Hanoi Medical University, Hanoi 115–20, Vietnam; kimbaogiang@hmu.edu.vn; 7 Center for Assessment and Quality Assurance, Hanoi Medical University, Hanoi 115–20, Vietnam; 8 Department of Psychiatry, Thai Nguyen University of Medicine and Pharmacy, Thai Nguyen 241–17, Vietnam; baohoaydtn@gmail.com; 9 Department of Pharmacognosy—Traditional Pharmacy—Pharmaceutical Botanic, Can Tho University of Medicine and Pharmacy, Can Tho 941–17, Vietnam; ltvtuyen@ctump.edu.vn; 10 Ph.D. Program in Clinical Drug Development of Herbal Medicine, College of Pharmacy, Taipei Medical University, Taipei 110–31, Taiwan; 11 Health Management Training Institute, Hue University of Medicine and Pharmacy, Thua Thien Hue 491–20, Vietnam; ntpthao.hmti@huemed-univ.edu.vn; 12 Department of Health Economics, Corvinus University of Budapest, Budapest 1093, Hungary; 13 Faculty of Public Health, Pham Ngoc Thach University of Medicine, Ho Chi Minh 725–10, Vietnam; nguyenthanhhiep@pnt.edu.vn; 14 Pham Ngoc Thach Clinic, Pham Ngoc Thach University of Medicine, Ho Chi Minh 725–10, Vietnam; 15 President Office, Pham Ngoc Thach University of Medicine, Ho Chi Minh 725–10, Vietnam; 16 School of Medicine and Pharmacy, Vietnam National University, Hanoi 113–09, Vietnam; thanhtrungnguyen.smp@gmail.com; 17 Faculty of Medical Laboratory Science, Da Nang University of Medical Technology and Pharmacy, Da Nang 502–06, Vietnam; ltthuy@dhktyduocdn.edu.vn; 18 President Office, Da Nang University of Medical Technology and Pharmacy, Da Nang 502–06, Vietnam; 19 Faculty of Nursing, Hai Duong Medical Technical University, Hai Duong 031–17, Vietnam; dohienhmtu@gmail.com; 20 Research Center of Geriatric Nutrition, Taipei Medical University, Taipei 110–31, Taiwan; 21 Nutrition Research Center, Taipei Medical University Hospital, Taipei 110–31, Taiwan

**Keywords:** COVID-19, coronavirus, health literacy, digital healthy diet literacy, psychometric properties, eating behavior, nursing student, medical student, online survey, Vietnam

## Abstract

Assessing healthy diet literacy and eating behaviors is critical for identifying appropriate public health responses to the COVID-19 pandemic. We examined the psychometric properties of digital healthy diet literacy (DDL) and its association with eating behavior changes during the COVID-19 pandemic among nursing and medical students. We conducted a cross-sectional study from 7 April to 31 May 2020 at 10 public universities in Vietnam, in which 7616 undergraduate students aged 19–27 completed an online survey to assess socio-demographics, clinical parameters, health literacy (HL), DDL, and health-related behaviors. Four items of the DDL scale loaded on one component explained 71.32%, 67.12%, and 72.47% of the scale variances for the overall sample, nursing, and medical students, respectively. The DDL scale was found to have satisfactory item-scale convergent validity and criterion validity, high internal consistency reliability, and no floor or ceiling effect. Of all, 42.8% of students reported healthier eating behavior during the pandemic. A 10-index score increment of DDL was associated with 18%, 23%, and 17% increased likelihood of healthier eating behavior during the pandemic for the overall sample (OR, 1.18; 95%CI, 1.13, 1.24; *p* < 0.001), nursing students (OR, 1.23; 95%CI, 1.10, 1.35; *p* < 0.001), and medical students (OR, 1.17; 95%CI, 1.11, 1.24; *p* < 0.001), respectively. The DDL scale is a valid and reliable tool for the quick assessment of digital healthy diet literacy. Students with higher DDL scores had a higher likelihood of healthier eating behavior during the pandemic.

## 1. Introduction

The unprecedented coronavirus disease (COVID-19) pandemic has created vast socioeconomic burdens [1], morbidity, and mortality [2,3]. The pandemic has also changed eating behaviors for the worse [4,5,6,7]. To deal with this challenge, the USA National Institute of Health Nutrition Research Task Force developed a 2020–2030 strategic plan to improve health and prevent or combat diseases and conditions that have been affected by food and nutrition [8].

Healthy dietary intake has shown protective effects on the immune systems and health outcomes during the COVID-19 crisis [9,10]. There are no data available, and rigorous clinical trials conducted to elaborate and confirm the benefits of a healthy diet in the current COVID-19 pandemic. Diversified and balanced diets improve the immune response to viral infection (e.g., SARS-CoV-2) [11], helping to reduce the severity [12] and any complications of COVID-19 [13]. Yet, unhealthy eating behaviors rapidly increased worldwide [14,15], including in Vietnam [16]. In ASEAN countries, the proportions of disordered eating attitudes among university students were 10%–20.6% [17].

The COVID-19 pandemic has also caused an “infodemic” of diverse information and sources [18,19]. Health literacy [20] and eHealth literacy [21] are highly recommended as strategic approaches to contain the pandemic. Nursing and medical students were mobilized to respond to the global health crisis [22,23,24,25], and because these future health workers can help combat misinformation and disinformation [26,27], their digital literacy should be promoted [28,29]. 

It is critical to promote healthy dietary behaviors to improve health outcomes [30] and contain the COVID-19 disease and its consequences [31,32,33]. The assessments of eating behaviors and nutritional status are critical to identifying comprehensive approaches for managing COVID-19 [34] and suggesting sustainable food intake [35]. To identify behavior changes and develop strategies to improve students’ eating behaviors, their ability to access, understand, appraise, and apply the healthy diet information on the internet during the pandemic requires a short, valid, and reliable survey tool. 

We examined the psychometric properties of an expanded digital healthy diet literacy (DDL) domain of health literacy to investigate associations between DDL and eating behavior changes during the COVID-19 pandemic among nursing and medical students at 10 public universities in Vietnam.

## 2. Materials and Methods

### 2.1. Study Design 

We conducted a cross-sectional online survey from 7 April to 31 May 2020. Participants from 10 universities across Vietnam were recruited, including those from six universities in the nation’s north, two in the south, and two in the country’s central region. The study settings were all public universities that train and provide the nation’s healthcare workforce.

### 2.2. Study Sample

Undergraduate medical and nursing students were invited as they are the medical university’s major focus and the country’s future healthcare providers. The data collection procedure was similar to a previous study [36]. In brief, researchers as university lecturers invited students to voluntarily participate, and there were no direct benefits or compensations for participating in the survey. Lecturers sent the survey link to student leaders via email, Messenger, and Zalo. The student leaders sent the link to other students. The online consent forms were signed before taking the survey. Students filled in the form with their name and phone number to avoid duplications, while data were coded and analyzed confidentially.

A total sample of 7616 students (out of 32,632 possible participants) aged 21 to 27 years completed the survey. The studied and possible students at each university are listed in Table 1.

### 2.3. Instruments and Measurements

#### 2.3.1. Sociodemographics and Clinical Parameters

Students reported sociodemographic information, including age, gender, academic year (1 to 6 for the medical field, 1 to 4 for nursing field), academic field (medical vs. nursing), and ability to pay for medication (very or fairly difficult vs. very or fairly easy).

Clinical parameters were assessed including self-reported body height (cm) and weight (kg). Body mass index (BMI, kg/m^2^) was also calculated. Students also reported health problems that resembled symptoms of COVID-19, which were recorded as suspected COVID-19 symptoms (S-COVID-19-S) [37]. These included common symptoms of fever, cough, and dyspnea along with less common symptoms of myalgia, fatigue, sputum production, confusion, headache, sore throat, rhinorrhea, chest pain, hemoptysis, diarrhea, and nausea/vomiting. If students had any of those symptoms, they were classified as having S-COVID-19-S or symptoms like COVID-19. Chronic health problems were assessed using Charlson comorbidity index items [38,39].

#### 2.3.2. Health Literacy and Digital Healthy Diet Literacy

Health literacy (HL) was evaluated using a 12-item short-form health literacy questionnaire (HLS-SF12) that has been widely used in Asian countries [40], including in Vietnam [41,42,43]. The HLS-SF12 questionnaire was used to measure comprehensive health literacy, including four stages of information processing (e.g., accessing, understanding, appraising, and applying), and three domains of health (e.g., health care, disease prevention, and health promotion) [44,45].

During the COVID-19 pandemic, digital healthy diet literacy (DDL) guides people towards healthier eating behaviors that can improve immune resistance [9,10]. Therefore, as an expanded concept of health literacy, DDL refers to the ability to access, understand, judge, and apply digital healthy-diet-related information to improve healthy eating behavior and health outcomes that are critical to contain the pandemic. We adapted the HL conceptual framework and expanded a DDL domain by adding four more items to assess the information processing ability, including the ability to (1) … find reliable and accurate healthy diet information on the internet, (2) … understand healthy diet information and dietary guidelines on the internet, (3) … judge whether healthy diet information on the internet is applied for individuals, and (4) … apply healthy diet information from the internet into individuals’ daily lives to eat healthily.

Participants reported difficulty in performing each task item based on 4-point Likert scales from 1 = very difficult to 2 = fairly difficult, 3 = fairly easy, and 4 = very easy. The HL and DDL indices were standardized to a unified metric from 0 to 50 with higher scores representing better HL or DDL [46] using Formula (1):

*Index* = (*Mean* − 1) × (50/3) (1)

where *Index* is the specific index calculated, *Mean* is the mean of all participating items for each individual, 1 is the minimal possible value of the mean (leading to a minimum value of the index of 0), 3 is the range of the mean, and 50 is the chosen maximum value. Both HLS-SF12 and DDL are subjective measures.

#### 2.3.3. Eating Behavior and Other Lifestyle Changes

Students reported their current health-related behaviors compared with before the pandemic [36]. We did not assess the actual dietary intake or eating habits in this study. Students simply self-reported the perception of eating behaviors. Students rated their eating behavior as less healthy, unchanged, and healthier. They also ranked their smoking, drinking, and physical activity on a scale ranging from never to stopped, less, unchanged, and more. During the pandemic, healthier eating, continued or additional physical activity, and decreases in or the cessation of both smoking and drinking are crucial, along with the risk management strategies of hand-washing, mask-wearing, and social distancing [31]. On the one hand, we did not investigate whether the “unchanged” eating behavior during the pandemic was considered “unhealthy” or “healthy” behavior. On the other hand, in health promotion, people are suggested to comply with healthy behaviors on every basis. These healthy behaviors are even more important during the pandemic; people should have healthier eating behaviors that can improve immune function and prevent viral infection. Therefore, we aimed to compare the “healthier diet” and other behavioral categories, including “unchanged” and “less healthy”. Similarly, because physical activity and cessation/reduction of smoking/drinking are suggested, “never, stopped, or less” were grouped into one category, and “unchanged or more” was another category. “Healthier diet” and “unchanged or more physical activity” were positive behaviors; “unchanged or more” smoking or drinking were negative behaviors. 

### 2.4. Ethical Consideration

The study was reviewed and approved by the Institutional Ethical Review Committee of Hanoi University of Public Health, Vietnam (IRB No. 133/2020/YTCC-HD3). Students voluntarily took the survey.

### 2.5. Data Analysis

#### 2.5.1. Psychometric Properties of Digital Healthy Diet Literacy

Principal component analysis (PCA) with the oblique rotation (Promax) method was utilized to assess the construct of the DDL scale. Correlations between the DDL scale and its four items were estimated using Spearman’s correlation test which provided evidence of item-scale convergent validity. In addition, the correlation between DDL and HLS-SF12 was estimated using the Pearson correlation test that provides evidence of criterion validity [47]. Cronbach’s alpha test was used to check the internal consistency of the DDL scale. The floor and ceiling effects of the DDL scale were assessed by calculating percentages of the possibly lowest and highest DDL index scores. 

#### 2.5.2. Health Literacy, Digital Healthy Diet Literacy, and Eating Behavior Changes

The frequency of eating behaviors (less healthy/unchanged vs. healthier) in different categories of socio-demographics, clinical parameters, and other health-related behaviors were explored using a Chi-square test. In addition, a one-way ANOVA test was used to check the distribution of HL and DDL scales by two categories of eating behavior. Multivariable logistic regression models were used to assess the associations of HL and DDL along with eating behavior change. Adjusted variables in the multivariable model were those demonstrating the associations with eating behavior change at *p* < 0.20 in the univariable analysis [48]. In order to exclude colliders which may cause multicollinearity, Spearman’s correlation test was used to check correlations between them. If the case of a moderate or high correlation, a representative variable was selected in the multivariable analysis.

Data analysis was conducted using IBM SPSS Version 20.0 for Windows (IBM Corp., Armonk, NY, USA). *p* < 0.05 was set for statistical significance. 

## 3. Results

### 3.1. Students’ Characteristics

The mean age was 21.4 ± 1.8 years. Out of all ages, 37.5% were men, 60.1% were 3rd–6th-year students, 75.7% were medical students, and 51.8% reported the ability to pay for medications at a very easy or fairly easy level. Of the study sample, 20.8% were underweight, 6.5% were overweight or obese, 19.2% reported S-COVID-19-S, and 4.4% reported one or more chronic health problems. In all, 2.9%, 6.3%, and 69.7% of students reported “unchanged or more” smoking, drinking, and physical activity during the pandemic as compared to that before the pandemic, respectively. During the pandemic, 42.8% of students reported healthier eating behavior compared to before the pandemic. Means of HL and DDL scores were 34.4 ± 6.9 and 33.9 ± 8.5, respectively (Table 2).

### 3.2. Psychometric Properties of Digital Healthy Diet Literacy

The Kaiser–Meyer–Olkin values (KMO) for the whole scale of the overall sample, nursing, and medical students were 0.78, 0.77, and 0.79, respectively. In addition, ranges of KMO values for individual items of the overall sample, nursing, and medical students were 0.75–0.83, 0.74–0.81, and 0.75–0.83, respectively. These values were higher than 0.6, which was set for measuring the sampling adequacy [49]. Furthermore, Bartlett’s Test of Sphericity values of the overall sample, nursing, and medical students were less than 0.05 which indicated data suitability for the PCA [49].

Four items were strongly loaded on a single component and explained 71.32%, 67.12%, and 72.47% of the scale variance in the overall sample, nursing, and medical students, respectively (Table 3). Ranges of correlations between the DDL scale and its four items were 0.80–0.83, 0.76–0.80, and 0.81–0.84 for the overall sample, nursing, and medical students, respectively. This provides adequate evidence of the item-scale convergent validity [50]. The DDL correlated with HL at a rho value of 0.68 for all, which provided satisfactory evidence of the criterion’s validity [47].

Cronbach’s alpha values of the DDL scale for the overall sample, nursing, and medical students were 0.86, 0.83, and 0.87, respectively, which were larger than 0.70, indicating the satisfactory reliability [51]. Percentages of the lowest and highest score in the overall sample (0.20% and 12.00%), nursing students (0.30% and 7.90%), and medical students (0.20% and 13.30%) were smaller than 15% which were reflected no floor and ceiling effects [52].

### 3.3. Associations of HL and DDL with Eating Behavior Changes

Age highly correlated with the academic year (rho = 0.95), gender moderately correlated with the academic field (rho = 0.36), and smoking moderately correlated with drinking (rho = 0.50; Table S1). Therefore, age, gender, and drinking were selected with medical payment ability, BMI, chronic health conditions, and physical activity for the overall sample; with BMI, and physical activity for the sample of nursing students; and with medical payment ability, chronic health conditions, and physical activities for the sample of medical students in the multivariable analysis. After adjusting for these mentioned confounders, a 10-score increment of HL and DDL indices were associated with the increased likelihood of healthier eating behavior of 23% and 18% for the overall sample, 24% and 23% for the sample of nursing students, and 23% and 17% for the sample of medical students (Table 4).

## 4. Discussion

The current study shows the digital healthy diet literacy (DDL) scale is a brief, valid, and reliable tool with satisfactory construct, criterion, and convergent validity, with a high level of internal consistency reliability, and with no floor or ceiling effect. The psychometric properties of the expanded DDL domain of HL were similar to the HLS-SF12 scale [40,41,53] and e-healthy diet literacy (e-HDL) scale [54]. Moreover, the performance of the DDL scale was similar in medical and nursing students. In a previous study, the e-HDL scale was developed with inconsistent response options for eleven survey questions. The number of questions was not equally distributed in different subscales [54]. Therefore, a shorter DDL questionnaire with the same concept and response options of the original health literacy that is needed for research and practice [44,45].

In this study, our findings illustrate that higher scores of health literacy and digital healthy diet literacy were positively associated with healthier eating behavior during the pandemic for both nursing and medical students. College students with higher eHealth literacy engaged better in positive health-promoting behaviors [29,55]. A previous study also showed that higher e-HDL scores were associated with better health status and health-related behaviors in the general population in Taiwan [54]. In addition, food literacy was found to be associated with healthier food consumption in the public in the Netherlands [56] and Canada [57]. In university settings, it is strategic to create opportunities (e.g., academic courses, media, and the internet) and motivators (e.g., social responsibility, and personal development) for students to develop food literacy and apply them in improving healthy eating behaviors and health outcomes [58]. Digital healthy diet literacy should be emphasized and taken into account for better decision making and health outcomes [59]. 

Medical school students play important roles in responding to the pandemic [22,23,60] and can promote healthy behaviors (e.g., healthy eating, preventive behaviors) to their peers, communities, and patients using social media or other available modalities [61,62]. Improving digital literacy focusing on a healthy diet for nursing and medical students is a strategic approach [28,29] that can influence public behavior in positive ways. 

We conducted an online cross-sectional study in which the findings cannot be interpreted as causal relationships. The category of “unchanged” eating behavior during the pandemic does not distinguish “unhealthy” from “healthy” behaviors that may potentially bias the analysis. In addition, we did not use questions about actual nutritional habits or behaviors and diet. The actual dietary intake was not assessed in the current study. Future studies are suggested to recruit questionnaires investigating dietary intake, such as a food frequency questionnaire or a 24 h food record form. Another limitation was that respondents were not informed about the meaning of “healthy eating behaviors” that may cause reporting bias and potentially affect the association between DDL and eating behaviors. Furthermore, the test–retest reliability of the DDL scale was not evaluated. However, the findings from the large sample analysis can possibly be generated to nursing and medical students. Despite the limitations, the online survey is suggested in conducting the study with fewer resources required and providing timely evidence for research and practice, further contributing to COVID-19 containment. Future studies are suggested to compare DDL, eating behaviors, and their association in students with those in general populations and healthcare workers. A longitudinal design is recommended to assess the long-term effect and causality.

## 5. Conclusions

The digital healthy diet literacy scale with four items (DDL-4) was an extended domain of a comprehensive health literacy framework. This scale was found to be a valid and reliable tool for the quick assessment of students’ ability to access, understand, appraise, and apply healthy diet information found on the internet. Nursing and medical students with higher HL or DDL scores had a higher likelihood of healthier eating behavior. The findings of our study provide a brief, valid, and reliable tool for research and practice during and after the COVID-19 pandemic.

## Figures and Tables

**Table 1:** Study participants in different universities by geographic locations.

Geographic Location	Hospital/Health Center	Academic Field	Possible Participants	Studied Participants
North				
Ha Noi	1. Hanoi Medical University	Medical	3386	844
		Nursing	285	166
	2. Vietnam Military Medical University	Medical	3034	1198
	3. Vietnam National University-School of Medicine and Pharmacy	Medical	444	385
Thai Nguyen	4. Thai Nguyen University of Medicine and Pharmacy	Medical	2830	742
		Nursing	579	200
Hai Duong	5. Hai Duong Medical Technical University	Nursing	697	379
Hai Phong	6. Haiphong University of Medicine and Pharmacy	Medical	3153	800
		Nursing	317	145
Center				
Thua Thien Hue	7. Hue University of Medicine and Pharmacy	Medical	3800	425
		Nursing	594	265
Da Nang	8. Da Nang University of Medical Technology and Pharmacy	Nursing	718	311
South				
Ho Chi Minh	9. Pham Ngoc Thach University of Medicine	Medical	5510	473
		Nursing	424	203
Can Tho	10. Can Tho University of Medicine and Pharmacy	Medical	6580	898
		Nursing	281	182
Subtotal		Medical	28,737	5765
		Nursing	3895	1851
Total			32,632	7616

**Table 2:** Participants’ characteristics and eating behavior change in the overall sample, nursing, and medical students.

Eating Behavior	Overall Sample				Nursing Students				Medical Students			
	Total (*N* = 7616)	Unchanged or Less Healthy (*N* = 4353)	Healthier (*N* = 3263)		Subtotal (*N* = 1851)	Unchanged or Less Healthy (*N* = 924)	Healthier (*N* = 927)		Subtotal (*N* = 5765)	Unchanged or Less Healthy (*N* = 3429)	Healthier (*N* = 2336)	
	*n* (%)	*n* (%)	*n* (%)	*p* *	*n* (%)	*n* (%)	*n* (%)	*p* *	*n* (%)	*n* (%)	*n* (%)	*p* *
Age, year				0.002				0.946				0.024
19–20	2834 (37.2)	1554 (35.7)	1280 (39.2)		957 (51.7)	477 (51.6)	480 (51.8)		1877 (32.6)	1077 (31.4)	800 (34.2)	
21–27	4782 (62.8)	2799 (64.3)	1983 (60.8)		894 (48.3)	447 (48.4)	447 (48.2)		3888 (67.4)	2352 (68.6)	1536 (65.8)	
Gender				<0.001				0.016				<0.001
Women	4762 (62.5)	2512 (57.7)	2250 (69.0)		1723 (93.1)	847 (91.7)	876 (94.5)		3039 (52.7)	1665 (48.6)	1374 (58.8)	
Men	2854 (37.5)	1841 (42.3)	1013 (31.0)		128 (6.9)	77 (8.3)	51 (5.5)		2726 (47.3)	1764 (51.4)	962 (41.2)	
Academic year				<0.001				0.756				0.004
Year 1–2	3036 (39.9)	1655 (38.0)	1381 (42.3)		1000 (54.0)	496 (53.7)	504 (54.4)		2036 (35.3)	1159 (33.8)	877 (37.5)	
Year 3–6	4580 (60.1)	2698 (62.0)	1882 (57.7)		851 (46.0)	428 (46.3)	423 (45.6)		3729 (64.7)	2270 (66.2)	1459 (62.5)	
Academic field				< 0.001								
Nursing	1851 (24.3)	924 (21.2)	927 (28.4)									
Medical	5765 (75.7)	3429 (78.8)	2336 (71.6)									
Ability to pay for medication				0.078				0.385				0.034
Very or fairly difficult	3674 (48.2)	2138 (49.1)	1536 (47.1)		1005 (54.3)	511 (55.3)	494 (53.3)		2669 (46.3)	1627 (47.4)	1042 (44.6)	
Very or fairly easy	3942 (51.8)	2215 (50.9)	1727 (52.9)		846 (45.7)	413 (44.7)	433 (46.7)		3096 (53.7)	1802 (52.6)	1294 (55.4)	
BMI, kg/m^2^				0.027				0.172				0.240
BMI < 18.5	1584 (20.8)	864 (19.9)	720 (22.1)		589 (31.8)	296 (32.0)	293 (31.6)		995 (17.2)	568 (16.6)	427 (18.3)	
18.5 ≤ BMI < 25.0	5535 (72.7)	3188 (73.3)	2347 (72.0)		1217 (65.8)	600 (64.9)	617 (66.6)		4318 (75.0)	2588 (75.5)	1730 (74.1)	
BMI ≥ 25.0	492 (6.5)	298 (6.9)	194 (5.9)		44 (2.4)	28 (3.1)	16 (1.7)		448 (7.8)	270 (7.9)	178 (7.6)	
S-COVID-19-S ^**^				0.361				0.705				0.283
No	6156 (80.8)	3503 (80.5)	2653 (81.3)		1461 (78.9)	726 (78.6)	735 (79.3)		4695 (81.4)	2777 (81.0)	1918 (82.1)	
Yes	1460 (19.2)	850 (19.5)	610 (18.7)		390 (21.1)	198 (21.4)	192 (20.7)		1070 (18.6)	652 (19.0)	418 (17.9)	
Comorbidity				0.029				0.576				0.021
None	7279 (95.6)	4141 (95.1)	3138 (96.2)		1762 (95.2)	877 (94.9)	885 (95.5)		5517 (95.7)	3264 (95.2)	2253 (96.4)	
One or more	337 (4.4)	212 (4.9)	125 (3.8)		89 (4.8)	47 (5.1)	42 (4.5)		248 (4.3)	165 (4.8)	83 (3.6)	
Smoking status				0.003				0.375				0.009
Never, stopped, or smoke less	7395 (97.1)	4205 (96.6)	3190 (97.8)		1818 (98.2)	905 (97.9)	913 (98.5)		5577 (96.7)	3300 (96.2)	2277 (97.5)	
Unchanged or smoke more	221 (2.9)	148 (3.4)	73 (2.2)		33 (1.8)	19 (2.1)	14 (1.5)		188 (3.3)	129 (3.8)	59 (2.5)	
Drinking status				<0.001				0.002				<0.001
Never, stopped, or drink less	7137 (93.7)	4005 (92.0)	3132 (96.0)		1791 (96.8)	882 (95.5)	909 (98.1)		5346 (92.7)	3123 (91.1)	2223 (95.2)	
Unchanged or drink more	479 (6.3)	348 (8.0)	131 (4.0)		60 (3.2)	42 (4.5)	18 (1.9)		419 (7.3)	306 (8.9)	113 (4.8)	
Physical activity				<0.001				<0.001				<0.001
Never, stopped, or exercise less	2309 (30.3)	1600 (36.8)	709 (21.7)		499 (27.0)	311 (33.7)	188 (20.3)		1810 (31.4)	1289 (37.6)	521 (22.3)	
Unchanged or exercise more	5307 (69.7)	2753 (63.2)	2554 (78.3)		1352 (73.0)	613 (66.3)	739 (79.7)		3955 (68.6)	2140 (62.4)	1815 (77.7)	
HL index, mean ± SD	34.4 ± 6.9	34.0 ± 6.9	34.9 ± 6.9	<0.001	34.4 ± 6.9	34.0 ± 6.9	34.9 ± 6.9	<0.001	34.4 ± 6.9	34.0 ± 6.9	34.9 ± 6.9	< 0.001
DDL index, mean ± SD	33.9 ± 8.5	33.3 ± 8.6	34.6 ± 8.4	<0.001	33.9 ± 8.5	33.3 ± 8.6	34.6 ± 8.4	<0.001	33.9 ± 8.5	33.3 ± 8.6	34.6 ± 8.4	< 0.001

* Result of one-way ANOVA test or Chi-square test appropriately. ** Suspected COVID-19 symptoms included common symptoms (fever, cough, dyspnea) and less common symptoms (myalgia, fatigue, sputum production, confusion, headache, sore throat, rhinorrhea, chest pain, hemoptysis, diarrhea, and nausea/vomiting).

**Table 3:** Construct, convergent, criterion validity, internal consistency, floor, and ceiling effects of digital healthy diet literacy scale (*N* = 7616).

DDL Scale	Overall Sample	Nursing Students	Medical Students
Factor loadings: “On a scale from very difficult to very easy, to what extent would you say it is difficult or easy to: …”			
1. Find reliable and accurate healthy diet information on the internet?	0.85	0.82	0.86
2. Understand healthy diet information and dietary guidelines on the internet?	0.88	0.86	0.88
3. Judge whether healthy diet information on the internet applied to you?	0.86	0.84	0.86
4. Apply healthy diet information from the internet to your daily life to make you eat better?	0.79	0.76	0.80
Percentage of variance, %	71.32	67.12	72.47
Item-scale convergent validity, mean of Rho (range) *	0.81 (0.80–0.83)	0.78 (0.76–0.80)	0.82 (0.81–0.84)
Criterion validity, correlation with HL, Rho **	0.68	0.68	0.68
Internal consistency, Cronbach’s alpha	0.86	0.83	0.87
Floor effects, %	0.20	0.30	0.20
Ceiling effect, %	12.00	7.90	13.3

* Rho, Spearman’s correlation coefficient. ** Rho, Pearson correlation coefficient.

**Table 4:** Associations of health literacy and digital healthy diet literacy with eating behavior changes (*N* = 7616).

Healthier Eating Behaviors *	Total Sample		Nursing Students		Medical Students	
	OR (95%CI)	*p*	OR (95%CI)	*p*	OR (95%CI)	*p*
HL index **						
Model 1	1.19 (1.12, 1.26)	<0.001	1.28 (1.13, 1.43)	<0.001	1.19 (1.12, 1.27)	<0.001
Model 2	1.23 (1.15, 1.30)	<0.001	1.24 (1.09, 1.40)	0.001	1.23 (1.15, 1.31)	<0.001
DDL index **						
Model 1	1.17 (1.11, 1.22)	<0.001	1.25 (1.13, 1.37)	<0.001	1.16 (1.10, 1.22)	<0.001
Model 2	1.18 (1.13, 1.24)	<0.001	1.23 (1.10, 1.35)	<0.001	1.17 (1.11, 1.24)	<0.001

* Reference group is “Eat less healthy or unchanged”. ** With a 10-index score increment. Model 1: Associations of the HL and DDL indexes with eating behavior change. Model 2: Adjusted for age, gender, ability to pay for medication, body mass index, comorbidity, drinking, physical activity for overall sample; adjusted for age, gender, body mass index, drinking, physical activity for the sample of nursing students; adjusted for age, gender, ability to pay for medication, comorbidity, drinking, physical activity for the sample of medical students.

## Supplementary Materials

The following are available online at https://www.mdpi.com/1660-4601/17/19/7185/s1, Table S1: Spearman’s correlation among the studied variables (*N* = 7616).

## Abbreviations

BMI	body mass index
COVID-19	coronavirus disease-2019
S-COVID-19-S	suspected coronavirus disease-2019 symptoms
SD	standard deviation
HL	health literacy
HLS-SF12	a 12-item short-form health literacy survey questionnaire
DDL	digital healthy diet literacy
OR	odds ratio
CI	confidence interval
