# Supplementary material for: Digital Healthy Diet Literacy and Self-Perceived Eating Behavior Change during COVID-19 Pandemic among Undergraduate Nursing and Medical Students: A Rapid Online Survey
Source: Int J Environ Res Public Health. 2020 Sep 30;17(19):7185. doi: 10.3390/ijerph17197185 (PMC7579441; doi:10.3390/ijerph17197185)
Supplement: Supplementary file 1 [file ijerph-17-07185-s001.pdf]

Table 1. Spearman's correlation among the studied variables (N = 7616).

	Age	Gender	Ability to Pay for Medication	Academic Year	Academic Field	BMI	S-COVID-19-S	Chronic Condition	Smoking	Drinking	Physical Activity
Gender	0.013										
Ability to pay for medication	−0.016	0.031									
Academic year	0.945	0.010	−0.003								
Academic field	0.170	0.358	0.069	0.164							
BMI	0.032	0.269	0.047	0.036	0.171						
S-COVID-19-S	−0.043	−0.021	−0.036	−0.046	−0.027	−0.015					
Chronic condition	0.015	0.026	0.002	0.010	−0.011	0.014	0.116				
Smoking	0.031	0.104	−0.010	0.029	0.038	0.031	0.001	0.016			
Drinking	0.060	0.101	−0.006	0.058	0.071	0.037	0.007	0.023	0.497		
Physical activity	−0.024	−0.041	0.047	−0.024	−0.041	−0.011	−0.034	−0.001	0.041	0.041	
HL index	0.146	0.098	0.154	0.144	0.066	0.036	−0.083	−0.020	0.052	0.018	0.063
DDL index	0.018	0.097	0.108	0.011	0.031	0.033	−0.083	−0.021	0.055	0.016	0.053

Abbreviations: BMI, body mass index; S-COVID-19-S, suspected coronavirus disease-2019 symptoms; HL, health literacy; DDL, digital healthy diet literacy.
